# Body mass index adjusted aspirin dosing does not overcome pharmacokinetic and pharmacodynamic disadvantages in obese pregnant women at risk for preeclampsia: a prospective cohort study

**DOI:** 10.3389/fmed.2026.1810134

**Published:** 2026-05-29

**Authors:** Fulya Sultan Karaduman, Numan Cim, Meltem Caliskan, Bünyamin Cim, Seval Yilmaz Ergani, Filiz Yarsilikal Guleroglu, Ali Cetin

**Affiliations:** 1 Department of Obstetrics and Gynecology, Haseki Training and Research Hospital, University of Health Sciences, Istanbul, Türkiye; 2 Department of Obstetrics and Gynecology, Kovancilar State Hospital, Elazig, Türkiye; 3 Department of Obstetrics and Gynecology, Department of Perinatology, Etlik City Hospital, University of Health Sciences, Ankara, Türkiye

**Keywords:** aspirin resistance, obesity, pharmacokinetics, preeclampsia, pregnancy, thromboxane B2, uterine artery Doppler

## Abstract

**Objective:**

To assess if aspirin dosed based on a patient's body mass index (BMI) would yield similar pharmacokinetics and pharmacodynamics for obese compared to non-obese patients who are taking aspirin to prevent preeclampsia.

**Methods:**

This prospective cohort study included 35 pregnant women at high-risk of developing preeclampsia from Haseki Training and Research Hospital, Istanbul, Türkiye. Participants were prospectively stratified (non-randomized) into two groups based on baseline BMI: Group 1 (*n* = 17, BMI <30 kg/m^2^, 100 mg/day aspirin) and Group 2 (*n* = 18, BMI ≥30 kg/m^2^, 150 mg/day aspirin). Blood salicylate levels, 11-dehydrothromboxane B2 (11-dTxB2) levels, uterine artery Doppler measurements, and diastolic notch presence were evaluated at both the baseline (weeks 12–16 of pregnancy) and follow up (weeks 16–20 of pregnancy). Biomarker analyses were available for 17 of 18 obese participants due to one missing sample.

**Results:**

Although the obese group was administered 50% more aspirin, they had 17% lower serum salicylate levels at follow-up (mean ± SD 2.08 ± 0.23 vs. 2.52 ± 0.32 μg/mL, *p* < 0.001). Although the 100 mg group had a significant decrease in 11-dTxB2 (*p* = 0.004), the 150 mg group did not have a significant decrease (*p* = 0.379). BMI had a strong inverse correlation with salicylate levels (r = −0.648, *p* < 0.001). A multiple regression model was developed using study group (β = 16.368, *p* = 0.002) and BMI (β = −1.984, *p* = 0.003) as the independent variables, and the uterine artery resistance index (UtA-RI) as the dependent variable. Mediation analysis showed that 21.0% of the total effect was numerically attributable to salicylate levels; however, the indirect effect was not statistically significant (Sobel *p* = 0.524). Uterine artery resistance change was the most significant predictor of diastolic notch resolution (OR = 0.86, *p* = 0.009).

**Conclusion:**

In this non-randomized cohort, a 150 mg aspirin dose did not appear to overcome the pharmacokinetic and pharmacodynamic disadvantages associated with obesity in pregnant women. Other dosing approaches, such as weight-based dosing or twice-daily administration, should be evaluated to optimize preeclampsia prophylaxis in obese pregnancies. These findings require confirmation in larger randomized trials.

## Introduction

Preeclampsia remains one of the leading causes of maternal and perinatal morbidity and mortality worldwide, affecting approximately 2–8% of pregnancies globally (1, 2). Preeclampsia presents a variety of clinical issues for obstetric care providers. The primary cause of preeclampsia is an inappropriate form of placentation and inadequate remodeling of the maternal spiral arteries that occur during the first trimester, resulting in insufficient oxygen delivery to the placenta; this triggers the release of anti-angiogenic factors, causing widespread damage to the vascular endothelium (3–5).

Low-dose aspirin is a first-line drug for the treatment of high risk women for the prevention of preeclampsia. The ASPRE trial was the first to show low-dose aspirin (150 mg) when started before the 16th week of pregnancy can decrease the rate of preterm preeclampsia by 62% in high-risk pregnant women based on results from their combined screening during the first trimester (6). Subsequent analyses confirm that the efficacy of aspirin is related to the dose administered; as a result, at doses ≥100 mg, aspirin's effect on the prevention of preeclampsia appears to be greater than at lower doses (7–9). In addition, the process by which aspirin prevents preeclampsia is due to its ability to irreversibly inhibit cyclooxygenase-1 (COX-1) in platelets, thus decreasing thromboxane A2 (TXA2), but not affecting prostacyclin production from the endothelium (10, 11).

Although there is strong evidence that aspirin can prevent complications such as preeclampsia for many pregnant people who are at high risk, it appears that a significant number of those at high risk will develop preeclampsia even when they take aspirin. It is estimated that approximately 20–30% of high-risk individuals may experience this problem (12, 13). This phenomenon of aspirin non-response or resistance has been attributed to multiple factors, including inadequate dosing, variable gastrointestinal absorption, and differences in platelet turnover rates. Recent pharmacokinetic studies have demonstrated that maternal body mass index (BMI) significantly influences aspirin bioavailability, with obese pregnant women exhibiting substantially lower plasma salicylic acid exposure compared to non-obese women receiving the same dose (14, 15). Finneran et al. (16) found that class III obesity (BMI >40 kg/m^2^) was associated with dramatically reduced odds of achieving adequate thromboxane suppression.

Obesity is known to cause many physical changes that can impact how aspirin works. These include increasing the amount of space where the medication is distributed in the body (therefore lowering the concentration of the drug in each area), altering the rate at which food passes through the stomach and into the intestines (thereby potentially affecting the absorption of the medication), and changing how quickly the liver metabolizes medications (therefore potentially reducing the effectiveness of the medication) (17, 18). Obese patients also have a chronic inflammatory response that causes platelets to be activated at a faster rate than normal. Therefore, obese patients are likely to need more frequent dosing of aspirin in order to maintain enough COX-1 enzyme inhibition (19, 20). Petrucci et al. found that obese individuals had reduced responses to once-daily low dose aspirin with evidence of platelet activation occurring despite the use of aspirin (21).

Given the documented impact of obesity on aspirin pharmacokinetics, a logical clinical approach has been to increase the aspirin dose in obese women. The ASPREO trial demonstrated that higher aspirin doses (162 mg vs. 81 mg) may be more effective in obese pregnant women, with a 78% probability of benefit in reducing severe preeclampsia (22). However, whether a simple linear dose-adjustment approach actually achieves the intended pharmacological equivalence has not been systematically investigated.

The primary aim of this study was to assess if the adjusted dose of aspirin based on the patients' BMI (150 mg/day for those with BMI ≥30 kg/m^2^ vs. 100 mg/day for those with BMI <30 kg/m^2^) would produce similar pharmacokinetic and pharmacodynamic results when given to pregnant women taking aspirin for preeclampsia prevention. To measure these results, we analyzed salicylate levels in the patient's serum as a pharmacokinetic marker; 11-dehydrothromboxane B2 (11-dTxB2), which is a product of platelet COX-1 inhibition, as an indicator of the drug's ability to inhibit COX-1 and uterine artery Doppler index as an indicator of blood flow to the uterus. These parameters were assessed at two time points, once before treatment (12–16 weeks gestation) and again after treatment (16–20 weeks gestation) to allow us to compare the effect of treatment on both the groups and within each group. Our secondary objectives were to compare the patients' clinical and laboratory values prior to the initiation of aspirin therapy; to analyze the percentage of patients who showed resolution of the diastolic notch, which can be indicative of improved uteroplacental circulation; and to compare the patients' gestational age at delivery, birth weight, and neonatal Apgar scores.

## Methods

### Study design and setting

This prospective cohort study was conducted at the Department of Obstetrics and Gynecology, Haseki Training and Research Hospital, University of Health Sciences, Istanbul, Türkiye, between February and June 2025. The study protocol was approved by the Local Ethics Committee (approval number: 10-2025; approval date: January 22, 2025) and was conducted in accordance with the Declaration of Helsinki. Written informed consent was obtained from all participants before enrollment.

Women pregnant with a single fetus, 18–40 years of age, who were currently taking aspirin for the prevention of preeclampsia were screened for inclusion into this study at a gestational age of 12–16 weeks. Eligible women had to meet three inclusion criteria. First, they had to be taking aspirin for preeclampsia prevention that started prior to their 12th week of gestation. Second, they had to have an increased risk of developing preeclampsia based on either clinical risk factors or their first trimester screening results. Lastly, they had to be able to give informed consent. Women who were eligible for the study were excluded if they met one of the following exclusion criteria: multiple gestation, previously documented contraindications to aspirin therapy, hypertensive disorder during pregnancy, kidney disease, liver disease, autoimmune diseases that require anticoagulant therapy, or less than 80% self-reported adherence to their aspirin therapy.

### Aspirin dosing strategy

Participants were prospectively stratified by BMI, and aspirin dosing was adjusted accordingly. Although low-dose aspirin is commonly administered as 75–81 mg in some clinical settings, a dose of 100 mg was selected in this study to align with widely accepted European clinical practice and guideline recommendations, which generally define low-dose aspirin as 75–150 mg. Additionally, using a 100 mg dose allowed for more precise dose standardization and facilitated a clear proportional increase to 150 mg in obese women. From a pharmacodynamic perspective, doses around 100 mg have been shown to achieve more consistent platelet cyclooxygenase-1 (COX-1) inhibition compared to lower doses, particularly in populations with increased body mass, where aspirin bioavailability and antiplatelet response may be reduced. Therefore, selecting 100 mg as the baseline dose provided a rational and clinically relevant framework for evaluating BMI-adjusted aspirin dosing (6, 23).

### Biomarker measurements

Blood and urine samples were collected at baseline (12 to 16 weeks of gestation) and follow-up (16 to 20 weeks of gestation, approximately 4 weeks after baseline). Samples were obtained 2–3 h after aspirin ingestion to capture peak serum concentrations during steady-state conditions of chronic therapy. Serum salicylate concentrations were measured using high-performance liquid chromatography (HPLC) with a lower limit of quantification of 0.1 μg/mL. 11-dehydrothromboxane B2 (11-dTxB2), the stable metabolite of thromboxane A2, was measured using a validated enzyme-linked immunosorbent assay (ELISA) kit (Cayman Chemical, Ann Arbor, MI).

### Uterine artery Doppler assessment

Uterine artery Doppler examinations were performed at baseline and follow-up by experienced sonographers blinded to aspirin dosing. Measurements were obtained using a Samsung V8 ultrasound system (Samsung Medison Co., Ltd., Seoul, South Korea) equipped with a 1–7 MHz convex transabdominal probe (CA1-7A). Bilateral uterine arteries were identified at the level of the internal cervical os, and pulsatility index (PI) and resistance index (RI) were calculated from three consecutive uniform waveforms. Mean values of PI and RI from bilateral uterine artery measurements were calculated and reported as mean uterine artery pulsatility index (UtA-PI) and mean uterine artery resistance index (UtA-RI). The presence and laterality of early diastolic notching were recorded at both time points and categorized as none, unilateral, or bilateral.

### Statistical analysis

Continuous variables were expressed as mean ± standard deviation. Distribution normality was assessed by Shapiro-Wilk test. Between-group comparisons were performed using independent samples *t*-test or Mann-Whitney U test as appropriate. Within-group changes from baseline to follow-up were analyzed using paired *t*-test. Categorical variables were compared using chi-square test or Fisher's exact test. Percent change was calculated as [(follow-up – baseline) / baseline] × 100. Percent change values reported in descriptive tables were calculated at the individual participant level and then summarized as mean ± SD. Group-level percent change values shown in Table 1 reflect change in group means and are presented for descriptive comparison. Effect sizes were calculated using Cohen's d for between-group comparisons.

**Table 1:** Aspirin biomarker levels at baseline and follow-up (biomarker analysis population).

Biomarker	Group 1: ASA 100 mg (BMI <30, *n* = 17)	Group 2: ASA 150 mg (BMI ≥30, *n* = 17^*^)	Between-group *p*
Salicylate - Baseline (μg/mL)	2.42 ± 0.38	2.14 ± 0.30	0.023^*^
Salicylate - Follow-up (μg/mL)	2.52 ± 0.32	2.08 ± 0.23	<0.001^**^
Salicylate - Δ Change	+0.10 (+6.4%)	−0.06 (−2.2%)	—
Salicylate - Within-group *p*	0.367	0.237	—
11-dTxB2 - Baseline (pg/mL)	883 ± 206	1,016 ± 270	0.115
11-dTxB2 - Follow-up (pg/mL)	764 ± 193	949 ± 243	0.020^*^
11-dTxB2 - Δ Change	−119 (−13.5%)	−68 (−6.7%)	—
11-dTxB2 - Within-group p	0.004^**^	0.379	—

Data are presented as mean ± SD. The 100 mg group achieved significant within-group TxB2 reduction (*p* = 0.004), while the 150 mg group did not (*p* = 0.379). Δ = change from baseline. ^*^p < 0.05; ^**^p < 0.01. ^*^Biomarker data were available for 17 of 18 participants in Group 2.

Pearson correlation coefficients were used to assess bivariate relationships between continuous variables in the pooled cohort. As a sensitivity analysis, within-group correlations were also computed separately for each dose group; given the reduced sample size (*n* = 17–18 per group), these subgroup correlations are reported in the Supplementary material and should be interpreted with appropriate caution regarding statistical power. Multiple linear regression was performed to determine the independent predictors for uterine artery Doppler changes and thromboxane inhibition. Only variables that had a *p* value <0.10 during the univariate analysis were entered into the multiple linear regression models. As an exploratory analysis, mediation testing was conducted to evaluate whether serum salicylate levels mediated the relationship between study group and uterine artery resistance changes; the methodology and results are detailed in the Supplementary material. The Sobel test was used to determine the significance of the indirect effect. To determine which variables were associated with the resolution of diastolic notch, logistic regression was performed.

Sample size estimation was performed using G^*^Power version 3.1 based on Pearson correlation analysis. Assuming a medium-to-large effect size (r = 0.6), alpha level of 0.05, and statistical power of 80%, a minimum of 15 participants per group was required. Accounting for an anticipated 10% attrition rate, the target sample size was set at 17 participants per group, resulting in a total planned sample of 35 participants. The study was prospectively powered for biomarker-based mechanistic endpoints rather than clinical pregnancy outcomes. The *a priori* sample size estimation was based on correlation analysis; the multiple linear regression and mediation models should therefore be considered exploratory, as formal power calculations for these multivariate analyses were not performed and the sample size may be insufficient for stable regression estimates. This sample size is consistent with published mechanistic biomarker studies of aspirin pharmacology in pregnancy. A two-sided *p*-value <0.05 was considered statistically significant. Statistical analyses were performed using SPSS version 29.0 (IBM Corp., Armonk, NY, USA) for descriptive statistics, group comparisons, correlation, and regression analyses. Mediation analysis and effect size calculations were conducted using R version 4.5.2 (R Foundation for Statistical Computing, Vienna, Austria) with the “mediation” and “effectsize” packages. Figures were generated using Python 3.11 with matplotlib and seaborn libraries.

## Results

### Baseline characteristics

**Figure 1:**
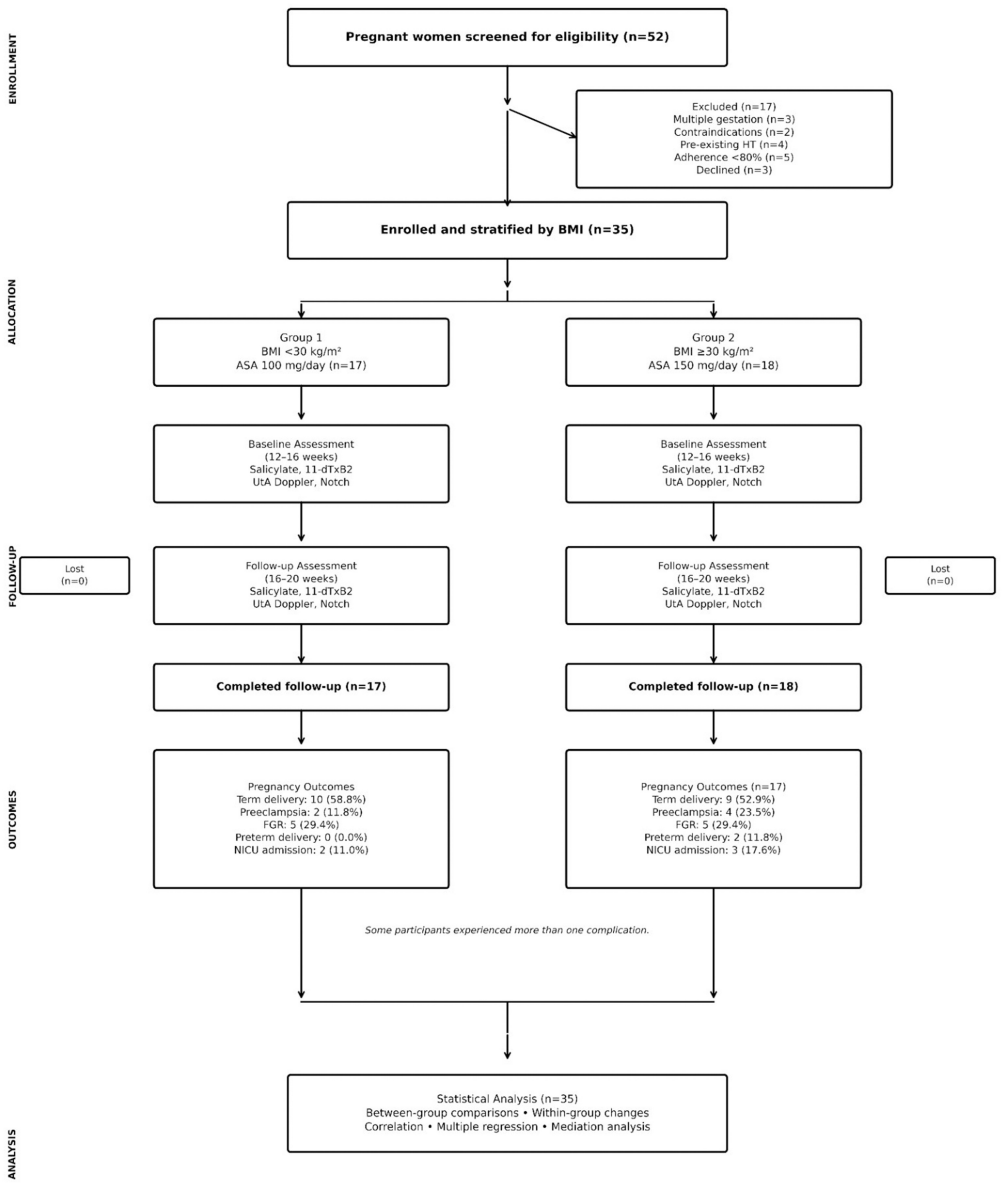
Participant flow through the study. Between February and June 2025, 52 pregnant women were screened for eligibility at Haseki Training and Research Hospital. Seventeen women were excluded due to multiple gestation (*n* = 3), contraindications to aspirin (*n* = 2), pre-existing hypertension (*n* = 4), self-reported adherence below 80% (*n* = 5), and declined to participate (*n* = 3). The remaining 35 women were enrolled and stratified by body mass index (BMI) into two groups: Group 1 (BMI <30 kg/m^2^, *n* = 17) receiving 100 mg aspirin daily and Group 2 (BMI ≥30 kg/m^2^, *n* = 18) receiving 150 mg aspirin daily. All participants completed baseline assessment (12–16 weeks of gestation) and follow-up assessment (16–20 weeks of gestation) with no loss to follow-up. Measurements included serum salicylate levels, 11-dehydrothromboxane B2, uterine artery Doppler parameters, and diastolic notch status. Pregnancy outcomes were recorded at delivery: In Group 1, term delivery occurred in 10 (58.8%), preeclampsia in 2 (11.8%), fetal growth restriction (FGR) in 5 (29.4%), preterm delivery in 0 (0.0%), and neonatal intensive care unit (NICU) admission in 2 (11.8%). In Group 2 (outcome data available for 17 participants; neonatal outcome records were missing for one enrolled participant), term delivery occurred in 9 (52.9%), preeclampsia in 4 (23.5%), FGR in 5 (29.4%), preterm delivery in 2 (11.8%), and NICU admission in 3 (17.6%). Some participants experienced more than one complication. Statistical analyses comprised between-group comparisons, within-group changes, Pearson correlation, multiple linear regression, and mediation analysis.

Between February and June 2025, 52 pregnant women were screened for eligibility at Haseki Training and Research Hospital. Seventeen women were excluded: multiple gestation (*n* = 3), contraindications to aspirin (*n* = 2), pre-existing hypertension (*n* = 4), self-reported adherence below 80% (*n* = 5), and declined to participate (*n* = 3). The remaining 35 women were enrolled and stratified by BMI into two groups: Group 1 (BMI <30 kg/m^2^, *n* = 17) receiving 100 mg aspirin daily, and Group 2 (BMI ≥30 kg/m^2^, *n* = 18) receiving 150 mg aspirin daily. All enrolled participants completed the baseline and follow-up assessments with no loss to follow-up (Figure 1).

The 100 mg group (*n* = 17) had a mean BMI of 25.5 ± 3.1 kg/m^2^, while the 150 mg group (*n* = 18) had a mean BMI of 31.6 ± 2.1 kg/m^2^ (*p* < 0.001). This significant difference in BMI was by design, as the study employed BMI-stratified dosing (BMI <30 kg/m^2^: 100 mg; BMI ≥30 kg/m^2^: 150 mg) rather than random dose allocation. The two groups were comparable in terms of age (30.1 ± 5.2 vs. 31.1 ± 6.9 years, *p* = 0.653), gestational age at enrollment (12.76 ± 0.76 vs. 12.76 ± 0.56 weeks, *p* = 0.803), and baseline laboratory parameters including hemoglobin, platelet count, white blood cell count, liver enzymes, and renal function (Table 2).

**Table 2:** Baseline demographic and clinical characteristics.

Variable	Group 1: ASA 100 mg (BMI <30, *n* = 17)	Group 2: ASA 150 mg (BMI ≥30, *n* = 18)	*p*-value
Age (years)	30.1 ± 5.2	31.1 ± 6.9	0.653
BMI (kg/m^2^)	25.5 ± 3.1	31.6 ± 2.1	<0.001^*^
Gravidity	2.0 (1.0–3.0)	2.0 (1.2–2.0)	0.756
Parity	1.0 (0.0–2.0)	1.0 (0.0–1.0)	0.670
Education level			0.988
Primary school, *n* (%)	4 (23.5%)	4 (22.2%)	
High school, n (%)	9 (52.9%)	10 (55.6%)	
University, n (%)	4 (23.5%)	4 (22.2%)	
Risk factors for preeclampsia			1.000
Previous hypertensive disease, *n* (%)	8 (47.1%)	8 (44.4%)	
Moderate risk factors combination, *n* (%)	9 (52.9%)	10 (55.6%)	
GA at enrollment (weeks)	12.76 ± 0.76	12.76 ± 0.56	0.803
Hemoglobin (g/dL)	12.0 ± 1.5	12.4 ± 0.9	0.393
Platelet count (103/μL)	254 ± 52	245 ± 68	0.682
WBC (103/μL)	9.6 ± 2.0	9.5 ± 2.2	0.809
AST (U/L)	15.2 ± 7.0	12.8 ± 3.6	0.454
ALT (U/L)	23.2 ± 10.2	16.8 ± 6.2	0.059
Creatinine (mg/dL)	0.58 ± 0.10	0.57 ± 0.04	0.665
GFR (mL/min/1.73 m^2^)	125.4 ± 15.1	124.1 ± 5.6	0.724

Data are presented as mean ± SD. The significant difference in BMI between groups was intentional, reflecting the study design. BMI, body mass index; GA, gestational age; WBC, white blood cell; AST, aspartate aminotransferase; ALT, alanine aminotransferase; ASA, acetylsalicylic acid; GFR, glomerular filtration rate. ^*^p < 0.05.

### Serum salicylate levels

**Figure 2:**
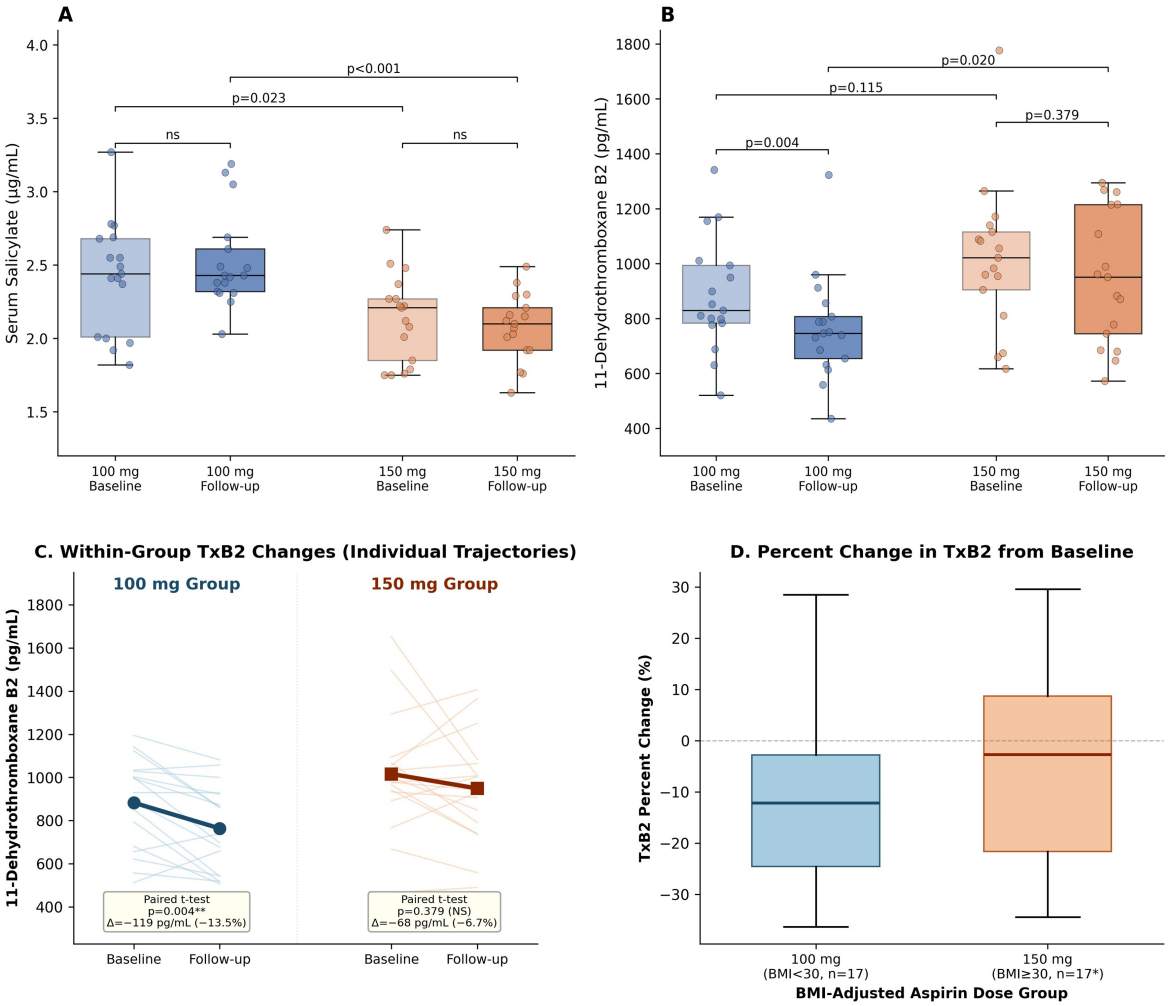
Aspirin biomarker responses (*n* = 17 per group*). Box plots with individual data points (jitter) showing median, interquartile range, and whiskers extending to 1.5 × IQR. **(A)** Serum salicylate levels. **(B)** 11-Dehydrothromboxane B2 levels. **(C)** Individual TxB2 trajectories. **(D)** Percent change in TxB2. *p < 0.05; **p < 0.01; ***p < 0.001. *Biomarker data were available for 17 of 18 participants in Group 2 due to one missing sample. Negative values in panel D indicate thromboxane suppression.

As prespecified, biomarker analyses were performed on 17 of 18 participants in Group 2 due to one missing blood sample (Table 1, Figure 2A). Within-group analyses confirmed that salicylate levels remained stable in both groups from baseline to follow-up (100 mg group: Δ = +0.10 μg/mL, *p* = 0.367; 150 mg group: Δ = −0.06 μg/mL, *p* = 0.237), consistent with steady-state conditions during chronic aspirin therapy. In descriptive between-group comparison, the 150 mg group had lower salicylate concentrations than the 100 mg group at both baseline (2.14 ± 0.30 vs. 2.42 ± 0.38 μg/mL, *p* = 0.023) and follow-up (2.08 ± 0.23 vs. 2.52 ± 0.32 μg/mL, *p* < 0.001), representing a 17% lower salicylate concentration in the higher-dose group with a very large effect size (Cohen's d = 1.60).

### 11 -Dehydrothromboxane B2 levels

The most notable finding emerged from the analysis of thromboxane inhibition (see Table 1 and Figures 2B–D). The 100 mg dose category had a significant decrease in 11-dTxB2 from 883 ± 206 pg/mL at baseline to 764 ± 193 pg/mL at follow-up (Δ = −119 pg/mL or −13.5%) as shown by a paired *t*-test with *p* = 0.004. This result shows statistically significant thromboxane suppression over the four week observation period. In contrast, the 150 mg dose category had no statistically significant decrease in 11-dTxB2 from 1016 ± 270 pg/mL at baseline to 949 ± 243 pg/mL at follow-up (Δ = −68 pg/mL or −6.7%), showing insufficient thromboxane suppression regardless of the fact that they received 50% more aspirin than the 100 mg dose category.

In descriptive between-group comparison, baseline 11-dTxB2 levels did not differ significantly (1016 ± 270 vs. 883 ± 206 pg/mL, *p* = 0.115). At follow-up, the between-group difference reached significance (949 ± 243 vs. 764 ± 193 pg/mL, p = 0.020, Cohen's d = −0.84), reflecting the widening gap produced by effective suppression in the 100 mg group and insufficient suppression in the 150 mg group.

### Uterine artery Doppler parameters

**Figure 3:**
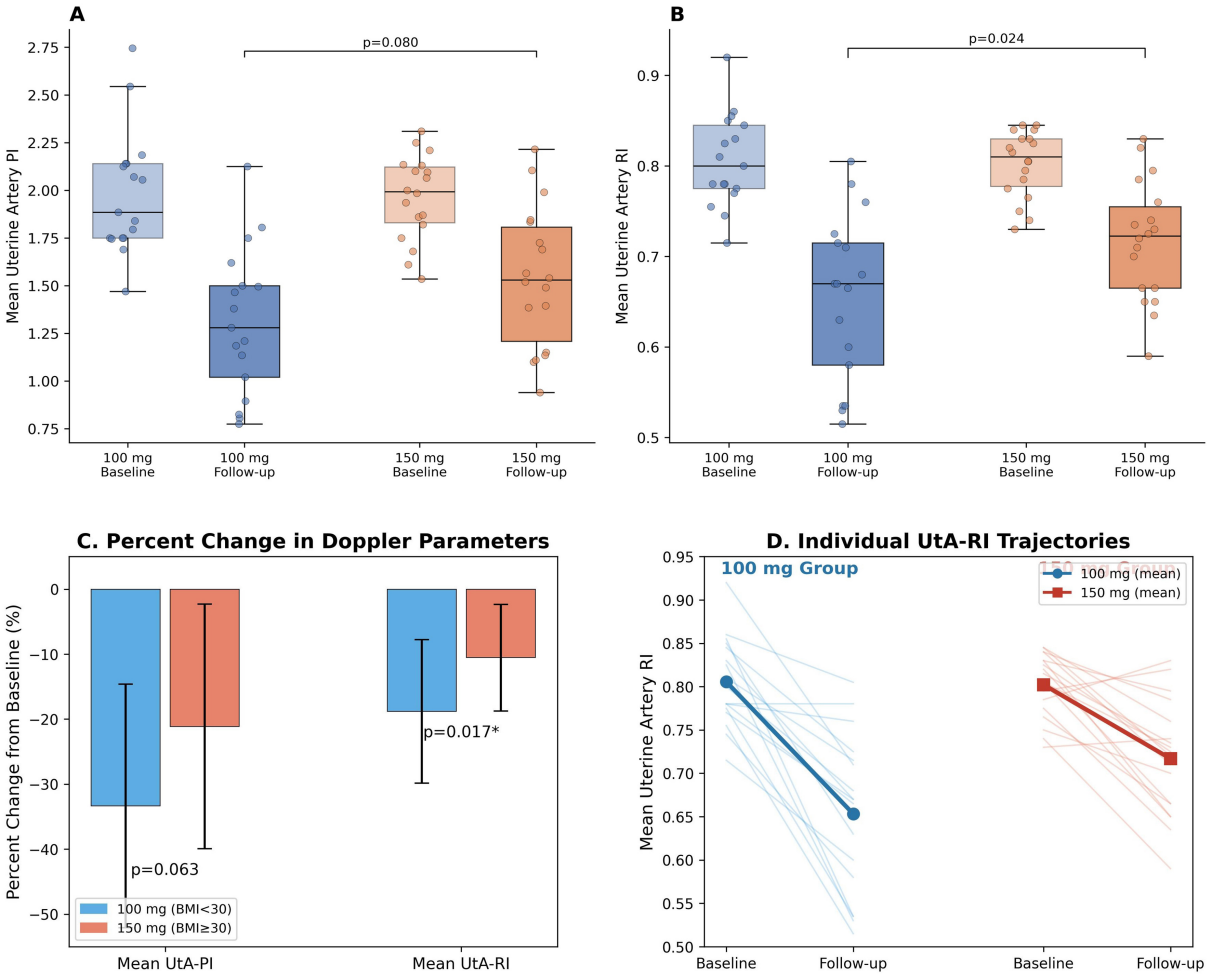
Uterine artery Doppler parameters. Box plots with individual data points (jitter) showing median, interquartile range, and whiskers extending to 1.5 × IQR. **(A)** Mean UtA-PI. **(B)** Mean UtA-RI. **(C)** Percent change. **(D)** Individual UtA-RI trajectories. *p < 0.05.

Baseline uterine artery Doppler parameters were comparable between groups (Table 3, Figure 3): mean UtA-PI was 1.98 ± 0.32 vs. 1.96 ± 0.22 (p = 0.849) and mean UtA-RI was 0.81 ± 0.05 vs. 0.80 ± 0.04 (*p* = 0.824). Both groups demonstrated within-group improvement from baseline to follow-up, consistent with expected gestational physiology. The magnitude of within-group improvement differed: the 100 mg group showed a mean UtA-RI percent change of −18.8 ± 11.0%, compared with −10.6 ± 8.2% in the 150 mg group (*p* = 0.017). In descriptive between-group comparison at follow-up, the 100 mg group had lower mean UtA-RI (0.65 ± 0.09 vs. 0.72 ± 0.07, *p* = 0.024) and a trend toward lower mean UtA-PI (1.31 ± 0.39 vs. 1.54 ± 0.37, *p* = 0.080).

**Table 3:** Uterine artery Doppler parameters.

Parameter	Group 1: ASA 100 mg (BMI <30, *n* = 17)	Group 2: ASA 150 mg (BMI ≥30, *n* = 18)	*p*-value
Mean UtA-PI - Baseline	1.98 ± 0.32	1.96 ± 0.22	0.849
Mean UtA-PI - Follow-up	1.31 ± 0.39	1.54 ± 0.37	0.080
Mean UtA-PI - % Change	−33.4 ± 18.7%	−21.1 ± 18.8%	0.063
Mean UtA-RI - Baseline	0.81 ± 0.05	0.80 ± 0.04	0.824
Mean UtA-RI - Follow-up	0.65 ± 0.09	0.72 ± 0.07	0.024^*^
Mean UtA-RI - % Change	−18.8 ± 11.0%	−10.6 ± 8.2%	0.017^*^

Data are presented as mean ± SD. The 100 mg group demonstrated significantly greater improvement in UtA-RI. UtA-PI, uterine artery pulsatility index; UtA-RI, uterine artery resistance index. ^*^p < 0.05.

### Uterine artery diastolic notch

At baseline, all participants exhibited abnormal uterine artery diastolic notching, with the majority showing bilateral notching (Table 4). In the 100 mg group, 13 participants (76.5%) had bilateral notching and 4 (23.5%) had unilateral notching. In the 150 mg group, 10 participants (55.6%) had bilateral notching and 8 (44.4%) had unilateral notching. At follow-up, both groups demonstrated substantial improvement in notch status. In the 100 mg group, 9 participants (52.9%) achieved complete notch resolution, 5 (29.4%) improved to unilateral, and 3 (17.6%) remained bilateral. In the 150 mg group, 10 participants (55.6%) achieved complete resolution, 6 (33.3%) improved to unilateral, and 2 (11.1%) remained bilateral. The overall notch improvement rates were similar between groups (70.6% vs. 77.8%, *p* = 0.711), as were the complete resolution rates (52.9% vs. 55.6%, *p* = 1.000).

**Table 4:** Uterine artery diastolic notch distribution and resolution.

Parameter	Group 1: ASA 100 mg (*n* = 17)	Group 2: ASA 150 mg (*n* = 18)	*p*-value
Baseline notch distribution			
Bilateral	13 (76.5%)	10 (55.6%)	0.289
Unilateral	4 (23.5%)	8 (44.4%)	
Follow-up notch distribution			
None (Resolved)	9 (52.9%)	10 (55.6%)	
Unilateral	5 (29.4%)	6 (33.3%)	0.932
Bilateral	3 (17.6%)	2 (11.1%)	
Notch outcomes			
Notch improvement rate	12/17 (70.6%)	14/18 (77.8%)	0.711
Complete resolution rate	9/17 (52.9%)	10/18 (55.6%)	1.000

Data are presented as *n* (%). Notch improvement defined as any reduction in notch severity. Complete resolution defined as absence of notching at follow-up.

### Correlation analysis

Bivariate correlation analysis revealed significant associations between key study variables (Table 5). BMI demonstrated a strong negative correlation with follow-up salicylate levels (r = −0.648, *p* < 0.001), confirming the pharmacokinetic disadvantage associated with obesity. Higher follow-up salicylate concentrations showed a near-significant association with greater TxB2 reduction (r = −0.337, *p* = 0.051), indicating that adequate drug exposure is necessary for effective thromboxane inhibition. Similarly, follow-up salicylate levels correlated negatively with UtA-RI percent change (r = −0.342, *p* = 0.048), suggesting that higher salicylate concentrations are associated with greater improvement in uterine artery resistance. No significant correlation was observed between TxB2 percent change and UtA-RI percent change (r = 0.257, *p* = 0.142). Within-group correlation analyses are presented in Supplementary Table S1; as anticipated given the reduced subgroup sample sizes (*n* = 17–18), several associations that reached significance in the pooled analysis did not attain statistical significance within individual groups.

**Table 5:** Correlation and multiple linear regression analyses.

Correlation analysis	Coefficient		*p*-value
BMI vs. Follow-up salicylate	r = −0.648		<0.001^***^
Follow-up salicylate vs. TxB2 % change	r = −0.337		0.051
Follow-up salicylate vs. UtA-RI % change	r = −0.342		0.048^*^
TxB2 % change vs. UtA-RI % change	r = 0.257		0.142
**Multiple regression: UtA-RI % change**	β	**95% CI**	***p*** **-value**
Model statistics	R^2^ = 0.394	F = 6.51	0.002^**^
Study group	16.368	6.49 to 26.25	0.002^**^
BMI	−1.984	−3.23 to −0.74	0.003^**^
Follow-up Salicylate	−10.053	−21.83 to 1.73	0.092
**Multiple regression: TxB2 % change**	β	**95% CI**	***p*** **-value**
Model statistics	R^2^ = 0.345	F = 5.27	0.004^**^
Follow-up salicylate	−25.815	−50.28 to −1.35	0.039^*^
Baseline TxB2	−0.045	−0.07 to −0.02	0.003^**^
Study Group	3.967	−13.21 to 21.15	0.641

^*^p < 0.05; ^**^p < 0.01; ^***^p < 0.001.

### Multiple linear regression analysis

Multiple linear regression was performed to identify independent predictors of uterine artery and thromboxane responses (Table 5). In the model predicting UtA-RI percent change (R^2^ = 0.394, F = 6.51, *p* = 0.002), study group (β = 16.368, 95% CI: 6.49 to 26.25, *p* = 0.002) and BMI (β = −1.984, 95% CI: −3.23 to −0.74, *p* = 0.003) emerged as significant independent predictors. These findings indicate that membership in the obese/higher-dose group and higher BMI were independently associated with less favorable UtA-RI improvement, even after controlling for salicylate levels.

In the model predicting TxB2 percent change (R^2^ = 0.345, F = 5.27, *p* = 0.004), follow-up salicylate level (β = −25.815, 95% CI: −50.28 to −1.35, *p* = 0.039) and baseline TxB2 level (β = −0.045, 95% CI: −0.07 to −0.02, *p* = 0.003) were significant independent predictors. Of particular interest, study group was not a significant predictor (β = 3.967, *p* = 0.641) after controlling for salicylate concentration, suggesting that the between-group difference in thromboxane inhibition is mediated primarily through differences in salicylate exposure rather than a direct group effect.

### Mediation analysis

Exploratory mediation analysis evaluating whether serum salicylate levels mediated the relationship between study group and UtA-RI percent change did not confirm a significant indirect effect (Sobel test: z = 0.64, *p* = 0.524). Full mediation results, including path coefficients and the mediation model diagram, are presented in Supplementary Figure S1 and Supplementary Table S2. Of note, the mediation model uses simple bivariate regressions for each path (without BMI as a covariate), which accounts for the difference in coefficient magnitudes compared to the multivariate regression model in Table 5.

### Predictors of notch resolution

Among participants who achieved complete diastolic notch resolution (*n* = 19), the mean UtA-RI percent change was −19.2 ± 9.6%, compared to −9.1 ± 8.7% in those who did not achieve resolution (*n* = 16, *p* = 0.003). Salicylate levels and TxB2 changes did not differ significantly between these groups (Table 6). Logistic regression analysis identified UtA-RI percent change as a significant independent predictor of notch resolution (OR = 0.86, 95% CI: 0.76 to 0.96, *p* = 0.009), indicating that each 1% greater reduction in UtA-RI was associated with 14% higher odds of complete notch resolution. Given the limited sample size, this exploratory logistic model should be interpreted cautiously.

**Table 6:** Predictors of diastolic notch resolution.

Parameter	Notch resolved (*n* = 19)	Notch not resolved (*n* = 16)	*p*-value
UtA-RI % change	−19.2 ± 9.6	−9.1 ± 8.7	0.003^**^
Follow-up salicylate (μg/mL)	2.30 ± 0.40	2.30 ± 0.30	0.988
TxB2 % change	−6.3 ± 24.0	−9.7 ± 19.3	0.657
**Logistic regression**	**OR**	**95% CI**	***p*** **-value**
UtA-RI % change	0.86	0.76 to 0.96	0.009^**^
Follow-up salicylate	0.20	0.01 to 2.96	0.240

Data are presented as mean ± SD. OR, odds ratio; CI, confidence interval. ^**^p < 0.01.

### Pregnancy outcomes

Pregnancy outcome data were available for 34 participants (Table 7), as neonatal outcome records were missing for one enrolled participant (Table 7). The 100 mg group showed significantly higher 1-min Apgar scores (8.47 ± 0.87 vs. 7.76 ± 0.97, *p* = 0.021). No significant differences were observed in gestational age at delivery (38.0 ± 2.0 vs. 37.5 ± 3.4 weeks, *p* = 0.876) or birth weight (3020 ± 667 vs. 2934 ± 781 g, *p* = 0.877). Preeclampsia developed in 2 participants (11.8%) in the 100 mg group and 4 participants (23.5%) in the 150 mg group (p = 0.656). FGR occurred in 5 participants (29.4%) in each group (*p* = 1.000). Preterm birth occurred in 2 participants (11.8%) in the 150 mg group only (*p* = 0.485). NICU admission was required for 2 (11.8%) and 3 (17.6%) neonates in the 100 mg and 150 mg groups, respectively (*p* = 1.000). The study was not powered to detect differences in clinical pregnancy outcomes; these endpoints are reported descriptively and should not be interpreted as definitive efficacy comparisons.

**Table 7:** Pregnancy outcomes.

Outcome	Group 1: ASA 100 mg (*n* = 17)	Group 2: ASA 150 mg (*n* = 17)	*p*-value
Gestational age at birth (weeks)	38.0 ± 2.0	37.5 ± 3.4	0.876
Birth weight (g)	3020 ± 667	2934 ± 781	0.877
APGAR 1st min	8.47 ± 0.87	7.76 ± 0.97	0.021^*^
APGAR 5th min	8.71 ± 1.10	8.76 ± 1.15	0.801
Preeclampsia, *n* (%)	2 (11.8%)	4 (23.5%)	0.656
FGR, *n* (%)	5 (29.4%)	5 (29.4%)	1.000
Preterm birth, *n* (%)	0 (0%)	2 (11.8%)	0.485
NICU admission, *n* (%)	2 (11.8%)	3 (17.6%)	1.000
Vaginal delivery, *n* (%)	7 (41.2%)	6 (35.3%)	1.000
Male gender, *n* (%)	9 (52.9%)	11 (64.7%)	0.728

Data are presented as mean ± SD or *n* (%). Mann-Whitney U test for continuous variables; Fisher's exact test for categorical variables. The study was not powered to detect differences in clinical outcomes. Some participants experienced more than one complication. ^*^p < 0.05.

## Discussion

This prospective cohort study evaluated whether an aspirin dose adjustment based on body mass index would help prevent preeclampsia. Using a non-randomized BMI-stratified dosing design that reflects real-world clinical practice, women who were obese received a 50% higher aspirin dose (150 mg vs. 100 mg), to see if it would produce similar results as the normal dose in women who are not overweight or obese. However, the researchers found that although the women who received the increased dose took more aspirin, they did not achieve equivalent salicylate exposure. These results contradict prior assumptions that simply increasing the dose of aspirin will provide adequate protection from complications of pregnancy related to hypertension for obese participants.

The study also supports previous research indicating that the higher-BMI group experiences less absorption of salicylate when taking aspirin during pregnancy. In their study of the pharmacokinetics of aspirin in pregnant women, Boelig et al. showed that there was a negative association between baseline obesity and current body mass index (BMI), and peak plasma salicylic acid concentration when women received 81 mg of aspirin per day (15, 24). Similarly, Rood et al. (14) have shown that women who are obese and pregnant receive much lower plasma salicylic acid exposure than do women who are not obese and pregnant. Our data show that even though women in the high-dose group received 50% more aspirin, they still experienced significantly lower salicylate levels (17%) than women in the low-dose group. The very strong negative correlation we found between BMI and salicylate levels (r = −0.648; *p* < 0.001) demonstrates the strong inverse association between these two variables. The large effect size (d = 1.60) we observed for salicylate levels also shows that the pharmacokinetic difference between the two groups is not only statistically significant, but clinically relevant.

Several mechanisms may explain this persistent pharmacokinetic disadvantage in obese pregnant women. Increased volume of distribution in obesity expands the drug distribution compartment, which can reduce peak plasma concentrations for a given dose (18, 25). Altered gastrointestinal absorption due to delayed gastric emptying and modified intestinal motility may reduce bioavailability (17). Changes in hepatic first-pass metabolism, protein binding, and renal clearance associated with both obesity and pregnancy compound these effects (26). The pregnancy-related plasma volume expansion adds an additional layer of pharmacokinetic complexity (27).

The lack of thromboxane inhibition in the 150 mg group is the most clinically significant finding of this study, and notably, this observation derives from within-group (pre- to post-treatment) comparisons that are not subject to the between-group confounding inherent in the BMI-stratified design. Although the 100 mg group experienced a strong 13.5% reduction in 11-dTxB2 levels (*p* = 0.004), the 150 mg group showed only a non-significant 6.7% reduction (*p* = 0.379) in their 11-dTxB2 levels. Our multiple regression analysis also confirmed that salicylate concentration, rather than aspirin dose group, was the primary determinant of thromboxane inhibition (β = −25.815, *p* = 0.039). Therefore, achieving adequate drug exposure is critical to achieving clinical efficacy. The chronic inflammation exhibited in obese individuals leads to an increase in platelet turnover through the elevated production of pro-inflammatory cytokines such as interleukin-6 and tumor necrosis factor-alpha (20, 28). The rapid turnover of platelets creates a larger reservoir of newly formed, aspirin-naive platelets entering into the bloodstream between doses (21, 29).

The temporal trends in our biomarker data provide further insight into the mechanisms of action observed in both groups. Both study groups achieved pharmacokinetic steady state, based on stable salicylate concentrations at baseline and follow-up. However, there were differences in the pharmacodynamic trajectories. The 100 mg group demonstrated progressive suppression of thromboxane, whereas the 150 mg group failed to demonstrate any degree of thromboxane suppression. This disparity between the pharmacokinetic stabilization of salicylate concentrations and the failure to suppress thromboxane activity is indicative of inadequate COX-1 inhibition during the dosing interval when platelet turnover is increased; therefore, once-daily dosing may not be sufficient to ensure COX-1 inhibition throughout the 24 h dosing interval. The observed improvements in uterine artery Doppler measurements are consistent with the biomarkers measured and demonstrate functional evidence of the differential treatment effects. The observed findings are consistent with previous research that suggests that the suppression of thromboxane may play a role in improving uteroplacental hemodynamics (30).

The results from the mediation analyses provide insight into the pathways through which aspirin dosing influences uterine artery hemodynamics. Exploratory mediation analysis did not confirm that salicylate levels significantly mediated the group-UtA-RI relationship (Supplementary Figure S1; Sobel *p* = 0.524), likely reflecting insufficient power for this test. The persistence of a large direct effect suggests that obesity-related mechanisms beyond drug exposure—such as altered platelet reactivity, endothelial dysfunction, and chronic inflammation—also contribute to the differential treatment response.

What stands out is that both study groups had similar rates of diastolic notch resolution (52.9% vs. 55.6%) despite the differences noted above regarding biomarkers and uterine artery Doppler response. However, our analysis also showed that notch resolution was a strong predictor of the degree of UtA-RI improvement (OR = 0.86 per 1% decrease in UtA-RI, *p* = 0.009), but not study group or salicylate level. Therefore, the notch resolution appears to reflect the physiologic result of improved uteroplacental perfusion regardless of whether the mechanism of improvement was via the pathway of thromboxane inhibition. The fact that some obese women achieved notch resolution while having less-than-optimal biomarker responses may represent variability in the association between the degree of thromboxane inhibition and vascular adaptation.

Our findings have important implications for clinical practice in preeclampsia prevention. The current paradigm of simple linear dose adjustment appears inadequate for obese pregnant women. Several alternative strategies warrant investigation: weight-based dosing (mg/kg body weight) may provide more consistent drug exposure across different body weights; twice-daily dosing may overcome increased platelet turnover by ensuring more consistent COX-1 inhibition throughout the day (29); the ASPREO trial demonstrated that 162 mg aspirin showed benefit compared to 81 mg in obese women, and even higher doses may be required (22); and biomarker-guided titration using 11-dTxB2 or salicylate levels may optimize therapeutic response (31). These observations support evaluating split-dose regimens and biomarker-guided titration as testable hypotheses in adequately powered randomized trials. These results provide a biologically plausible rationale for randomized trials evaluating weight-adjusted or split-dose aspirin regimens in obese pregnancy.

There was no significant difference in the rate of pregnancy complications between the two groups as measured by obstetrics. Preeclampsia occurred at a similar rate in the 100 mg and 150 mg treatment groups, 11.8% (*n* = 2) and 23.5% (*n* = 4), respectively (*p* =0.656) and there was no statistically significant difference in the incidence of Fetal Growth Restriction (FGR) in either group, 29.4% in each group (*p* = 1.000). While preterm delivery was identified exclusively in the 150 mg aspirin treatment group, 11.8% (*p* = 0.485), the percentage of patients admitted to the Neonatal Intensive Care Unit (NICU) was equivalent in each group, 11.8% vs. 17.6% (*p* = 1.000). Although the study did not have enough power to assess for significant differences in clinical outcomes, the trend toward increased complication rates in the 150 mg aspirin treatment group despite receiving a higher aspirin dose is consistent with the pharmacokinetic and pharmacodynamic findings. The failure of a 50% dose increase to result in improved clinical outcomes supports the hypothesis that the pharmacologic disadvantages associated with obesity cannot be overcome simply through linear dose escalations. Lastly, the higher mean 1 min Apgar score in the 100 mg group (8.47 ± 0.87 vs. 7.76 ± 0.97, *p* = 0.021) may suggest better overall placental function and fetal wellbeing in the non-obese cohort; however, this finding should be viewed cautiously due to the small sample size.

## Strengths and limitations

In addition to a number of methodological advantages that contribute to the reliability of the results, this study has several strengths. As a prospective study with standardized measurements, the data collection process was consistent. By measuring pharmacokinetic and pharmacodynamic biomarkers (for example, thromboxane levels) at two different times, this study comprehensively measured how individuals respond to aspirin. The inclusion of uterine artery Doppler parameter measurements along with the measurement of diastolic notch provided a functional connection to the biochemical findings. Finally, the advanced statistical methodology employed by this study (including multiple regression and mediation analysis) permitted the identification of independent predictors of aspirin response as well as the underlying mechanisms associated with the effects of aspirin.

The results of this study should be viewed in the context of an exploratory mechanistic study. Therefore, while this study was conducted to investigate the mechanism(s) by which aspirin exerts its effects on blood coagulation, it was not intended to provide information regarding the clinical efficacy of aspirin in preventing pregnancy complications (such as preeclampsia). However, several limitations must be acknowledged. First, the relatively small sample size limits the power to detect differences among groups and may have contributed to the non-significant Sobel test statistic in the mediation analysis. Second, the random assignment of patients to treatment groups was not feasible due to the fact that aspirin dosing was based on institutional protocol and was determined by body mass index (BMI). While this was done to reflect typical clinical practices, the lack of randomization introduces the possibility of selection bias into the study design. Third, the inherent link between aspirin dose assignment and BMI means that between-group comparisons cannot disentangle the independent effects of dose vs. BMI, and residual confounding cannot be fully excluded. This is a fundamental constraint of the BMI-stratified dosing design. Accordingly, our between-group comparisons should be considered descriptive rather than causal; the within-group (pre- to post-treatment) changes, which are not subject to this confounding, provide stronger evidence of differential aspirin response. Fourth, as a single center study, the ability to generalize to other populations is likely limited. Fifth, we did not measure platelet aggregation directly nor assess platelet turnover rates which would have provided further insight into the biological mechanisms by which aspirin influences platelet activity. Sixth, the short duration of follow-up precludes the assessment of longer term pregnancy outcomes, such as the incidence of preeclampsia, for which the study was not adequately powered. Seventh, as the analyses conducted in this study were exploratory in nature and multiple comparisons were made, the results should be viewed with caution and validated in future larger studies. We evaluated variance inflation factors for each model in order to ensure there was no substantial multicollinearity in the regression models. Finally, we did not compare different aspirin formulations or investigate dose-response relationships within the obese cohort to determine the most appropriate aspirin formulation or dosage strategy.

## Conclusion

The results of this study suggest that BMI-adjusted aspirin dosing using a linear dose-escalation method does not appear to overcome the pharmacokinetic and pharmacodynamic disadvantages associated with obesity in pregnant women. Although obese patients received larger doses of aspirin than did non-obese patients, obese patients had less aspirin-induced salicylate exposure and also were found to have less thromboxane inhibition than their non-obese counterparts. These findings suggest that the pharmacological alterations associated with obesity may not be adequately addressed by a simple linear dose increase alone. Therefore, other methods of achieving effective aspirin prophylaxis in obese pregnant women, including weight-based dosing, use of two doses per day (split-dose regimen), or biomarker-assisted titration of aspirin dosage, need to be evaluated in prospective, adequately powered, randomized studies. In the meantime, clinicians should recognize that the currently accepted methods for administering aspirin are likely inadequate to ensure optimal aspirin prophylaxis for obese pregnant women, and these preliminary findings need to be confirmed by appropriately designed and powered clinical trials.

## Data Availability

The original contributions presented in the study are included in the article/Supplementary material, further inquiries can be directed to the corresponding author.
