# Supplementary material for: Body mass index adjusted aspirin dosing does not overcome pharmacokinetic and pharmacodynamic disadvantages in obese pregnant women at risk for preeclampsia: a prospective cohort study
Source: Front Med (Lausanne). 2026 May 29;13:1810134. doi: 10.3389/fmed.2026.1810134 (PMC13260331; doi:10.3389/fmed.2026.1810134)
Supplement: Supplementary file 1 [file Supplementary_file_1.docx]

**Supplementary Material**

*Body Mass Index Adjusted Aspirin Dosing Does Not Overcome Pharmacokinetic and Pharmacodynamic Disadvantages in Obese Pregnant Women at Risk for Preeclampsia: A Prospective Cohort Study*

**Supplementary Table S1.** Within-Group Pearson Correlation Analysis Compared with Pooled Cohort Correlations

| **Correlation** | **Pooled r** | **Pooled p** | **n** | **Group 1 r** | **Group 1 p** | **n** | **Group 2 r** | **Group 2 p** | **n** |
| --- | --- | --- | --- | --- | --- | --- | --- | --- | --- |
| BMI vs Follow-up Salicylate | -0.648 | <0.001 | 34 | -0.416 | 0.097 | 17 | -0.131 | 0.617 | 17 |
| Follow-up Salicylate vs TxB2 % Change | -0.337 | 0.051 | 34 | -0.202 | 0.437 | 17 | -0.362 | 0.153 | 17 |
| Follow-up Salicylate vs UtA-RI % Change | -0.342 | 0.048 | 34 | -0.135 | 0.604 | 17 | -0.076 | 0.771 | 17 |
| TxB2 % Change vs UtA-RI % Change | 0.257 | 0.142 | 34 | 0.098 | 0.709 | 17 | 0.286 | 0.265 | 17 |

*Group 1: 100 mg aspirin (BMI <30 kg/m²); Group 2: 150 mg aspirin (BMI ≥30 kg/m²). Within-group correlations are based on reduced sample sizes (n=17–18 per group) and should be interpreted with caution regarding statistical power.*

*Abbreviations: BMI, body mass index; TxB2, 11-dehydrothromboxane B2; UtA-RI, uterine artery resistance index.*

**Supplementary Table S2.** Mediation Analysis: Path Coefficients for the Relationship Between Study Group, Serum Salicylate, and UtA-RI Percent Change

| **Path** | **β (unstd.)** | **SE** | **p** | **Sobel z** | **Sobel p** |
| --- | --- | --- | --- | --- | --- |
| Path a (Group → Salicylate) | −0.446 | 0.102 | <0.001 | — | — |
| Path b (Salicylate → UtA-RI Δ%) | −4.020 | 6.280 | 0.524 | — | — |
| Path c (Total effect) | 8.543 | 3.380 | 0.016 | — | — |
| Path c’ (Direct effect) | 6.748 | 4.510 | 0.144 | — | — |
| Indirect effect (a × b) | 1.795 | — | — | 0.64 | 0.524 |

*The mediation model uses simple bivariate regressions for each path (without BMI as a covariate). Path a: effect of study group on mediator (salicylate). Path b: effect of mediator on outcome (UtA-RI % change) controlling for group. Path c: total effect of group on outcome. Path c’: direct effect controlling for mediator. The indirect effect (a × b = 1.795) did not reach statistical significance, indicating that formal mediation through salicylate levels could not be confirmed. The numerically estimated proportion mediated was 21.0%; however, this should be interpreted with caution given the non-significant indirect pathway and the limited sample size (n=35) for mediation testing.*

*Abbreviations: β, unstandardized regression coefficient; SE, standard error; UtA-RI, uterine artery resistance index.*


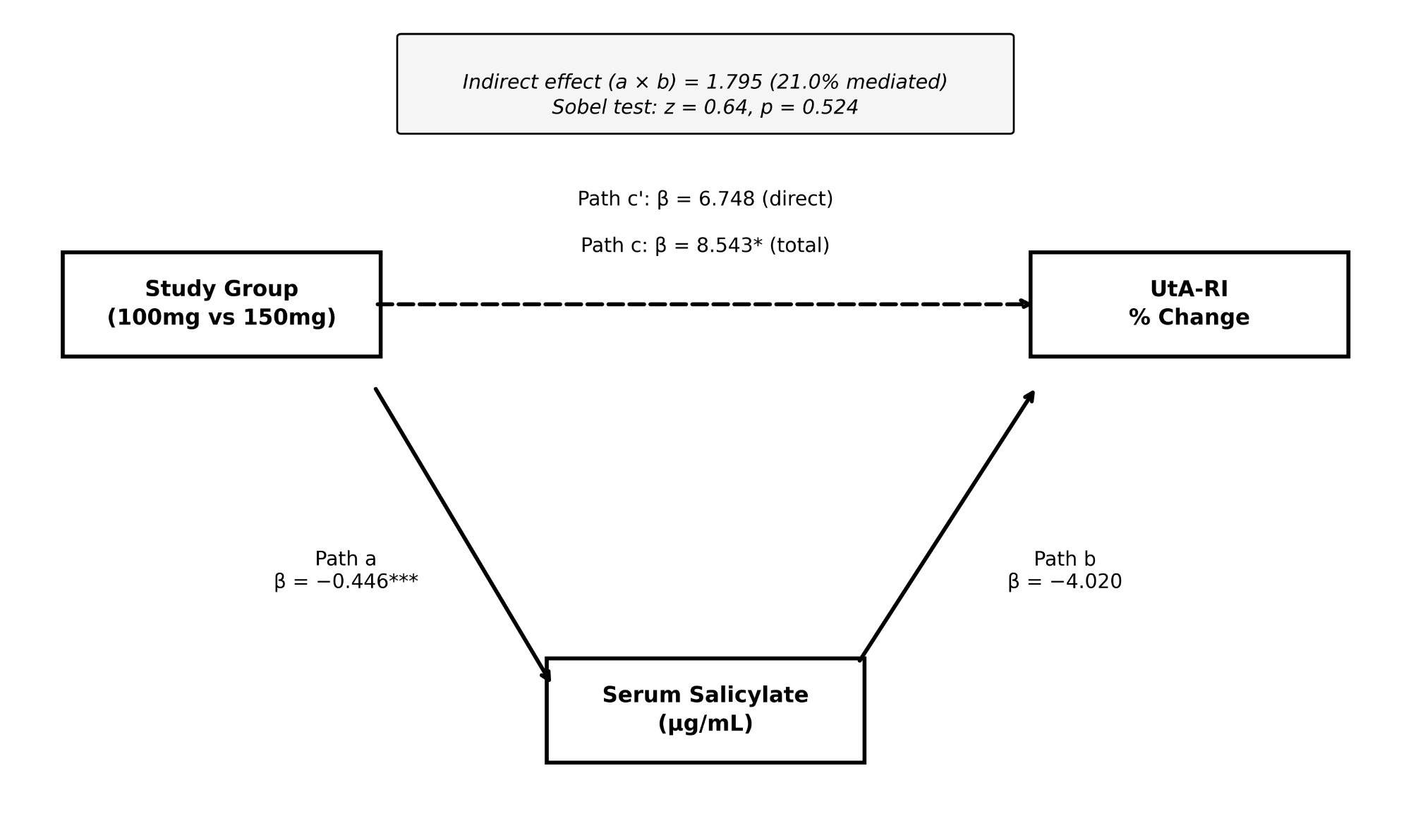


**Supplementary Figure S1.** Mediation model evaluating whether serum salicylate accounts for part of the relationship between study group and UtA-RI change. Path coefficients are unstandardized regression coefficients. The indirect effect was not statistically significant (Sobel test: z=0.64, p=0.524). ***p < 0.001; *p < 0.05. [Figure 4 from original submission is relocated here as Supplementary Figure S1.]
